# Lambda-Carrageenan Enhances the Effects of Radiation Therapy in Cancer Treatment by Suppressing Cancer Cell Invasion and Metastasis through Racgap1 Inhibition

**DOI:** 10.3390/cancers11081192

**Published:** 2019-08-16

**Authors:** Ping-Hsiu Wu, Yasuhito Onodera, Frances C. Recuenco, Amato J. Giaccia, Quynh-Thu Le, Shinichi Shimizu, Hiroki Shirato, Jin-Min Nam

**Affiliations:** 1 Global Station for Quantum Medical Science and Engineering, Global Institution for Collaborative Research and Education (GI-CoRE), Hokkaido University, Sapporo, Hokkaido 060-8638, Japan; 2 Department of Radiation Oncology, Graduate School of Medicine, Hokkaido University, Sapporo, Hokkaido 060-8638, Japan; 3 Department of Molecular Biology, Faculty of Medicine, Hokkaido University, Sapporo, Hokkaido 060-8638, Japan; 4 Department of Radiation Oncology, Stanford University School of Medicine, Stanford, CA 94305, USA; 5 Department of Radiation Oncology, Faculty of Medicine, Hokkaido University, Sapporo, Hokkaido 060-8638, Japan; 6 Research Center for Cooperative Project, Faculty of Medicine, Hokkaido University, Sapporo, Hokkaido 060-8638, Japan

**Keywords:** carrageenan, invasion, metastasis, RacGAP1, radiotherapy

## Abstract

Radiotherapy is used extensively in cancer treatment, but radioresistance and the metastatic potential of cancer cells that survive radiation remain critical issues. There is a need for novel treatments to improve radiotherapy. Here, we evaluated the therapeutic benefit of λ-carrageenan (CGN) to enhance the efficacy of radiation treatment and investigated the underlying molecular mechanism. CGN treatment decreased viability in irradiated cancer cells and enhanced reactive oxygen species accumulation, apoptosis, and polyploid formation. Additionally, CGN suppressed radiation-induced chemoinvasion and invasive growth in 3D lrECM culture. We also screened target molecules using a gene expression microarray analysis and focused on Rac GTPase-activating protein 1 (RacGAP1). Protein expression of RacGAP1 was upregulated in several cancer cell lines after radiation, which was significantly suppressed by CGN treatment. Knockdown of RacGAP1 decreased cell viability and invasiveness after radiation. Overexpression of RacGAP1 partially rescued CGN cytotoxicity. In a mouse xenograft model, local irradiation followed by CGN treatment significantly decreased tumor growth and lung metastasis compared to either treatment alone. Taken together, these results suggest that CGN may enhance the effectiveness of radiation in cancer therapy by decreasing cancer cell viability and suppressing both radiation-induced invasive activity and distal metastasis through downregulating RacGAP1 expression.

## 1. Introduction

Radiotherapy is a standard treatment to eliminate cancer cells by inducing a variety of cellular events, including the accumulation of reactive oxygen species (ROS) [1] and apoptotic cell death [2]. In clinical practice, radiotherapy is used as a post-operative treatment after resection, treatment for inoperable tumors, and may even replace surgery where organ preservation is desired [3,4]. Although radiotherapy is an effective anticancer therapy, differences in radiation response among different types of cancers [5,6], along with the side effects of high dose or large field IR [7], limit its efficacy. To increase its clinical impact, various chemotherapeutic drugs are administered with radiotherapy. However, these drugs can have their toxicity to normal tissues [8]. Therefore, novel therapies that are free from side effects but at the same time can improve radiotherapy by increasing cancer cell death and reducing distal metastasis are highly desirable.

Lambda-carrageenan (CGN), a family of linear sulfated polysaccharides, has diverse biological activities, which include anti-coagulant [9], anti-viral [10], and anti-tumor effects [11]. In addition, carrageenans are safely used as a food additive under FDA regulations in the United States [12]. Recently, several studies have reported the anti-tumor effects of CGN by stimulating an immune response in mice [11,13]. However, the effect of CGN in combination with IR on cancer treatment and its molecular mechanisms are not known. Considering the safety profile of this agent, we evaluated the effect of CGN as an adjuvant treatment to radiotherapy.

In several types of cancer, recent studies have identified upregulated expression of RacGAP1 (also known as MgcRacGAP or hCYK-4), a member of the guanine triphosphatase (GTPase) activation protein (GAP) family and suggested its potential role in promoting tumor progression [14,15,16]. RacGAP1 regulates the activation of Rho GTPase, which is reported to drive tumor growth [17] and to act as an oncogene in basal-like breast cancers [18]. Moreover, RacGAP1 is required for integrin-related invasive cell migration in the three-dimensional extracellular matrix (3D ECM) [19]. In clinical studies, RacGAP1 has attracted increasing attention as a predictive biomarker for metastasis and prognosis in several types of cancer [15,20].

In this study, we investigated the effect of CGN combined with ionizing radiation (IR) on cancer treatment and determined underlying molecular mechanisms. We found that CGN treatment after IR decreased cancer cell survival and invasiveness. Gene expression analysis showed that RacGAP1 was upregulated after IR treatment, and significantly suppressed by CGN treatment. Furthermore, CGN treatment after IR significantly suppressed tumor growth and lung metastasis in an in vivo model. These results indicate that CGN is a potential therapeutic adjuvant to radiotherapy, improving its therapeutic effect by suppressing RacGAP1.

## 2. Results

### 2.1. CGN Treatment Decreases the Cell Viability in Irradiated Cancer Cell Lines

**Figure 1:**
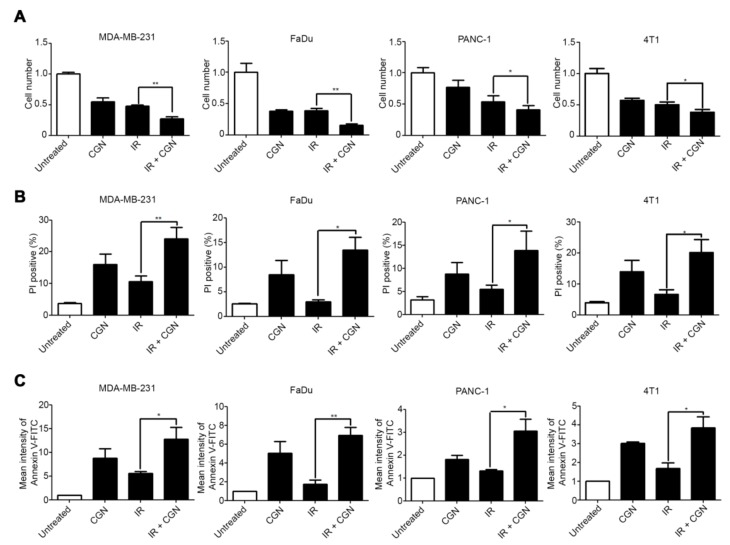
λ-carrageenan (CGN) treatment decreases cell viability and increases apoptosis in irradiated cancer cell lines. Cells were treated with 4 Gy IR, followed by CGN on the next day, and then analyzed 72 h after IR. (**A**) Cell viability was quantified by cell counting in MDA-MB-231, FaDu, PANC-1, and 4T1 cell lines. (**B**) The percentage of dead cells was measured by PI staining, followed by flow cytometry. The proportion of dead cells was quantified by gating the population of PI-positive cells. (*p*-values: MDA-MB-231, 0.0056; FaDu, 0.0129; PANC-1, 0.0489; 4T1, 0.0468.) (**C**) Apoptotic cells were measured by Annexin V-FITC staining, followed by flow cytometry. Mean fluorescence intensity of FITC was calculated and normalized to the untreated group. Columns, mean (*n* ≥ 3); bars, SE. *, *p* < 0.05; **, *p* < 0.01.

The possibility of using polysaccharides as an adjuvant to improve the anti-tumor efficacy of traditional chemotherapy has been raised [21,22]. In this study, we determined the anti-tumor effects of CGN combined with IR in the MDA-MB-231 breast cancer, FaDu head and neck cancer, PANC-1 pancreatic cancer, and 4T1 murine breast cancer cell lines. In each case, when compared to IR alone, cell number decreased significantly when IR and CGN were combined. The result also showed that CGN alone caused damage to cancer cells (Figure 1A). To further analyze the decrease in cell number in each condition, we next stained the cells with propidium iodide (PI) and Annexin V. CGN treatment following IR led to a significant increase in PI-positive cells (Figure 1B) and Annexin V-positive cells (Figure 1C) compared to the IR alone group. Annexin V/PI double staining analysis showed that late apoptosis was increased in cells treated with IR and CGN compared to those treated with IR alone (Figure S1). These results suggest that CGN combined with IR decreases viability in several cancer cell lines by induction of apoptotic cell death. We evaluated the effect of CGN and IR treatment in non-malignant epithelial cell line MCF10A (Figure S2A–C). The CGN-induced toxicity following IR treatment in MCF10A cells was not higher than that in other malignant cells.

### 2.2. IR Combined with CGN Treatment Increases ROS Accumulation in MDA-MB-231 Breast Cancer Cells

**Figure 2:**
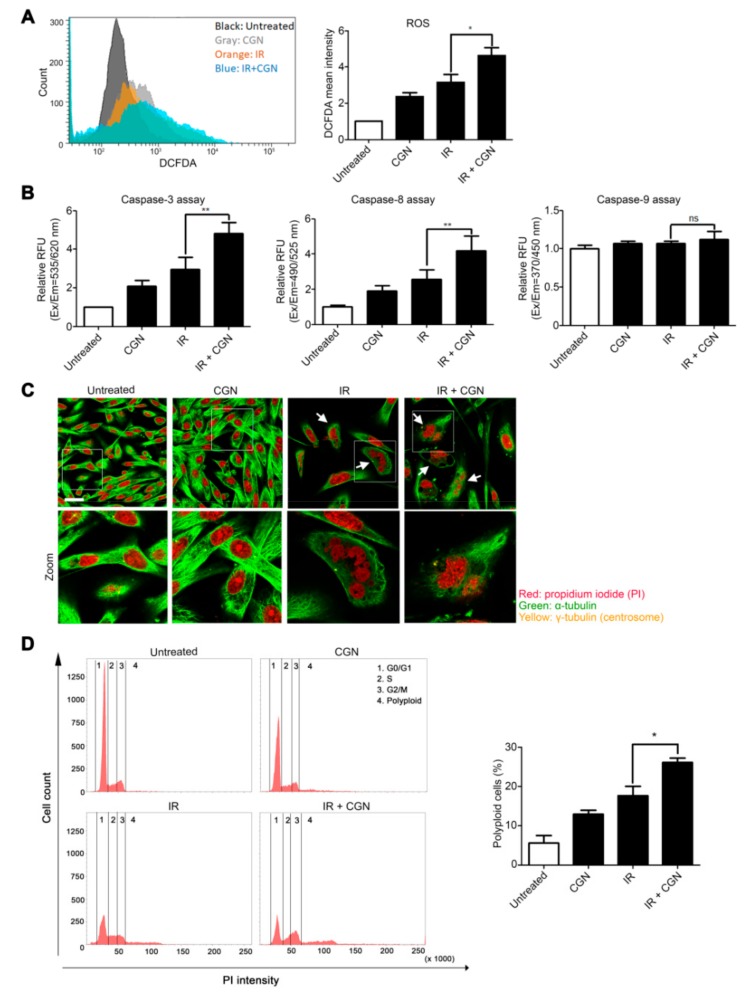
IR exposure in combination with CGN increases ROS accumulation in MDA-MB-231 cells. Cells were treated with 4 Gy IR, followed by CGN on the next day, and then analyzed 72 h after IR. (**A**) ROS was measured by DCFDA. Columns, mean (*n* = 5); bars, SE. *, *p* < 0.05. (**B**) Caspase-3, caspase-8, and caspase-9 activities were detected by microplate reader at specific wavelengths: caspase-3 excitation (Ex)/emission (Em) = 535/620 nm; caspase-8 Ex/Em = 490/525 nm; caspase-9 Ex/Em = 370/450 nm. Columns, mean (*n* = 5); bars, SE. **, *p* < 0.01; ns, not significant. (**C**) Cells stained with α-tubulin (green) and PI (red) after treatments. Bar, 25 μm. (**D**) To measure polyploid populations, cells were treated with staining solution and PI and analyzed by flow cytometry. Columns, mean (*n* = 3); bars, SE. *, *p* < 0.05.

Elevation of ROS is an important factor in the control of cancer cell death in radiotherapy [23]. It is known that IR induces ROS, which mediate apoptotic cell death and mitotic failure. Additionally, CGN has been reported to increase the production of ROS in human colonic epithelial cells [24]. We analyzed cellular ROS levels using DCFDA, which fluoresces when oxidized by ROS. Increased ROS levels were observed in the IR and CGN treated cells, compared to IR alone (Figure 2A). CGN or IR alone also showed an increase in ROS accumulation. High levels of ROS are known to activate caspase-3 and caspase-8, which are the key proteins of apoptosis [25]. The activities of caspase-3 and caspase-8, but not caspase-9, were elevated after IR followed by CGN in comparison to IR alone in MDA-MB-231 cells (Figure 2B). Consistent with these results, an increase in cleaved caspase-3 level in the IR and CGN treated cells, compared to the other groups, was also confirmed by western blot (Figure S3). These results indicate that apoptosis-related cell death is efficiently induced by CGN following IR, which is consistent with the PI and Annexin V staining (Figure 1).

Besides apoptotic cell death, IR is known to cause mitotic catastrophe [26,27], a mechanism of mitosis-linked cell death resulting in polyploid cell formation [28]. Generation of ROS is also reported to permit inappropriate entry into mitosis and induce mitotic catastrophe [29]. To determine whether mitotic catastrophe was induced by CGN combined with IR, we analyzed polyploid formation in the cells by immunofluorescence. Under confocal fluorescence microscopy, abnormal polyploid giant cells were observed in both the IR alone and combined treatment groups (Figure 2C). The proportion of polyploid cells was significantly increased by combined treatment with CGN and IR compared to IR alone (Figure 2D). These data suggest that CGN can increase ROS accumulation in irradiated cells, which may further enhance caspase-mediated apoptosis and mitosis-related cell death.

### 2.3. CGN Inhibits the Radiation-Induced Invasiveness of Breast Cancer Cell Lines

Cancer cells with high invasive capacity are correlated with poor prognosis [30,31]. Several groups have reported that failure of tumor control by IR could be associated with cancer invasiveness and subsequent distal metastasis [32,33], highlighting a potentially undesirable effect of radiotherapy. Our previous studies showed that cancer cell invasiveness could be increased in the surviving population after IR treatment through integrin-mediated pathways [34,35]. We, therefore, investigated CGN’s effect on the invasiveness of surviving cells after IR. The invasive activity was increased in the breast cancer cell lines after IR treatment, as we have reported previously [35]. 

**Figure 3:**
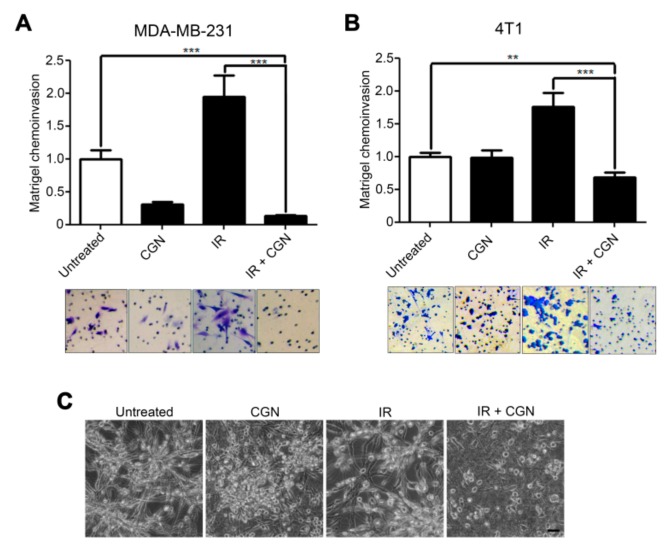
CGN inhibits the IR-induced invasive activity and 3D lrECM growth in breast cancer cells. (**A**, **B**) The invasive activity was measured by Matrigel chemoinvasion assay after IR and/or CGN treatments in MDA-MB-231 (**A**) and 4T1 (**B**) cells. Columns, mean (*n* = 4); bars, SE. **, *p* < 0.01; ***, *p* < 0.001. (**C**) MDA-MB-231 cells were cultured in 3D lrECM. Bar, 50 µm.

**Figure 4:**
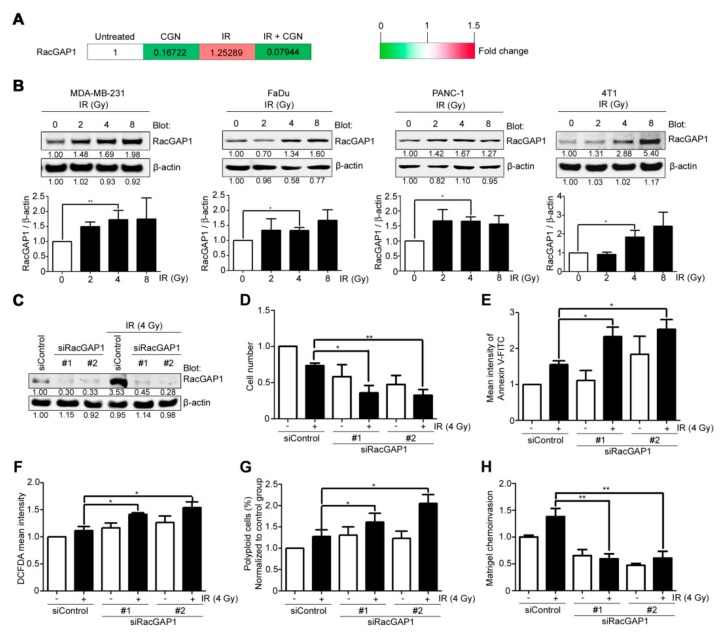
Upregulation of RacGAP1 is involved in cancer cell survival and invasion after IR. (**A**) Gene expression was analyzed by cDNA microarray. RacGAP1 expression level is shown by the heat map. The values were normalized to the untreated group. (**B**) RacGAP1 protein expressions with different doses of IR were analyzed in MDA-MB-231, FaDu, PANC-1, and 4T1 cell lines. (**C**) MDA-MB-231 cells were transfected with siRNA duplexes targeting RacGAP1 (# 1 or # 2) or the control sequence, as indicated. Cell lysates were subjected to western blot two days after transfection. (**D**–**H**) MDA-MB-231 cells were transfected with siRNAs and incubated for two days, and then treated with 4 Gy IR. Each experiment was performed 24 h after IR treatment. Cell viability was quantified by cell counting (**D**). Apoptotic cells were measured by Annexin V-FITC staining and flow cytometry (**E**). ROS was measured by DCFDA (**F**). Cells were treated with staining solution and PI, and then the cell cycle was analyzed by flow cytometry. The percentage of polyploid cells in each group was normalized with control group (**G**). The invasive activity was measured by Matrigel chemoinvasion assay (**H**). Columns, mean (*n* ≥ 3); bars, SE. *, *p* < 0.05; **, *p* < 0.01. Data shown in (**D**–**H**) were normalized to that of MDA-MB-231 cells transfected with siRNA control. For the continuous full length-image of Western blot signals, please refer to Supplementary Materials Figure S7.

Interestingly, the invasive ability of MDA-MB-231 (Figure 3A) and 4T1 (Figure 3B) breast cancer cell lines was significantly lower in the combined treatment with IR and CGN compared to IR alone. Cell viability was not affected during the invasion assay (Figure S4). These data indicate that CGN suppresses the IR-related invasiveness of these cells. Compared to the results of cytotoxicity depicted in Figure 1 and Figure 2, CGN showed a higher anti-invasive effect which is specific in post-IR cells. This effect was more obvious in MDA-MB-231 cells, which leads to a more significant reduction in invasive ability than that of CGN alone, suggesting the possibility that CGN suppresses specific mechanisms, which induced the increase in invasiveness in post-IR cancer cells. To further confirm the effect of CGN in the invasive growth of cancer cells, we performed 3D laminin-rich ECM (lrECM) culture. Culturing cells in 3D lrECM is a common method to assess the physiologically relevant morphogenesis and oncogenic properties of non-malignant or cancerous mammary epithelial cells [36]. As shown in Figure 3C, untreated MDA-MB-231 cells under 3D culture displayed aggressive invasive growth with stellate protrusions extending into the lrECM. The formation of protrusions was reduced in cancer cells that received the combination of CGN and IR treatment, indicating suppression of their invasive capacity in 3D lrECM culture.

### 2.4. Upregulation of RacGAP1 Is Involved in Cancer Cell Survival and Invasion after IR

To determine the effects on molecular pathways, differential gene expression in each treatment group was assessed by cDNA microarray. We selected the genes that were both upregulated by IR treatment and suppressed by the following CGN treatment (Figure S5). From these genes, we focused on RacGAP1 as a potential target (Figure 4A) because this protein is intimately connected with integrin signaling, which we have been mainly working on [34,35,37]. Moreover, RacGAP1 is recently reported to have important roles in oncogenic activity [19], tumor progression [15,16], and cancer invasion [38,39].

To investigate its role in irradiated cells, protein levels of RacGAP1 were determined after IR treatment in different types of cancer cell lines (Figure 4B). RacGAP1 was found to be upregulated after IR treatment, suggesting that it plays a role in the cellular response to radiation. Compared to the control group, knockdown of RacGAP1 by siRNA (Figure 4C and Figure S6) resulted in a significant decrease in cell viability following IR treatment (Figure 4D). Knockdown of RacGAP1 also increased the apoptosis, ROS accumulation, and polyploid formation after IR treatment in MDA-MB-231 cells (Figure 4E–G). Furthermore, knockdown of RacGAP1 effectively suppressed the IR-induced invasion activity of cancer cells (Figure 4H). These results indicate that RacGAP1 inhibition could increase the effectiveness of IR by reducing both cell viability and IR-induced invasiveness, which was also achieved by adding CGN after IR.

### 2.5. RacGAP1 Expression Is Suppressed by CGN in MDA-MB-231 Cells

**Figure 5:**
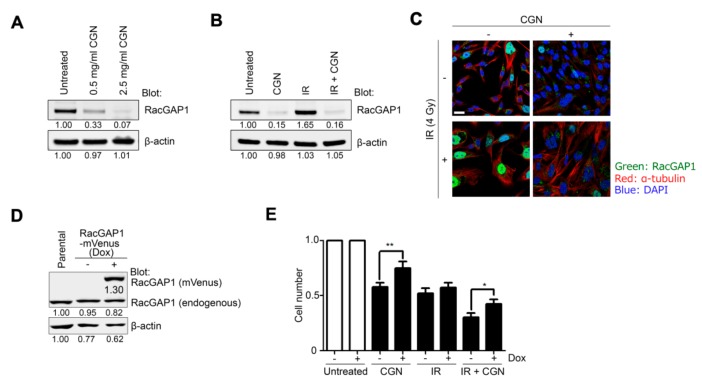
RacGAP1 expression is suppressed by CGN in MDA-MB-231 cells. (**A**) Protein expression of RacGAP1 was analyzed after treatment with different concentrations of CGN in MDA-MB-231 cells. Total cell lysates were subjected to western blot. (**B**) RacGAP1 protein expression was analyzed using cell lysates after IR and/or CGN treatment in MDA-MB-231 cells. (**C**) Immunofluorescence images show RacGAP1 (green), α-tubulin (red) and nuclei (blue). Bar, 25 μm. (**D**) RacGAP1-mVenus expression was induced by doxycycline in MDA-MB-231 cells. RacGAP1 expression was analyzed by western blot using anti-RacGAP1 antibody. Dox, doxycycline. (**E**) Cell number was quantified by cell counting. Columns, mean (*n* = 3); bars, SE. *, *p* < 0.05; **, *p* < 0.01. For the continuous full length-image of Western blot signals, please refer to Supplementary Materials Figure S7.

The cDNA microarray data suggest that CGN treatment suppresses RacGAP1 (Figure 4A). Consistent with the gene expression data, we also confirmed that the protein expression of RacGAP1 was significantly downregulated by CGN (Figure 5A). Moreover, elevation of RacGAP1 protein levels following IR, and its suppression after CGN treatment were also confirmed by western blot (Figure 5B). Immunofluorescent staining showed that RacGAP1 mainly localized in the nucleus and that its nuclear level was increased after IR and suppressed by CGN treatment (Figure 5C).

To further confirm that the downregulation of RacGAP1 is involved in the cytotoxic effect of CGN, we generated a doxycycline-inducible overexpression system of RacGAP1 (Figure 5D). RacGAP1 overexpression partially reduced the cytotoxicity caused by CGN with or without IR treatment (Figure 5E), which suggests that RacGAP1 is indeed an important molecular target in CGN treatment.

### 2.6. CGN in Combination with IR Decreases Tumor Size and Metastasis in a Mouse Xenograft Model

**Figure 6:**
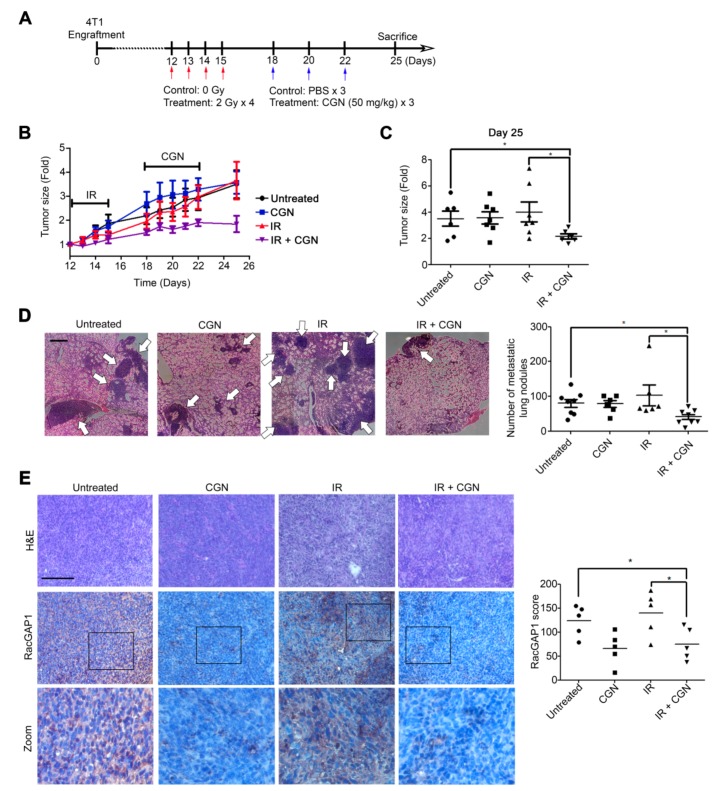
Radiation followed by CGN treatment decreases tumor size and metastasis in a 4T1 mouse xenograft model. (**A**) Treatment schedule of each group. Mouse 4T1 cells were injected into Balb/c mice (*n* = 6–7 in each group). (**B**) 4T1 tumor sizes were measured and normalized to size at day 12 in each group. (**C**) Relative tumor size for each group was measured on Day 25. Scatter plot; mean ± SE. *, *p* < 0.05. (**D**) Representative H&E images of lung sections. Bar, 500 µm. Arrow, metastatic lung nodules. The number of metastatic lung nodules was counted (right panel). Scatter plot; mean ± SE. *, *p* < 0.05. (**E**) Sections from 4T1 tumors were subjected to IHC staining with antibodies against RacGAP1. Bar, 100 µm. RacGAP1 expression level was determined by scoring, as described in the methods (right panel). Scatter plot; bars, mean (*n* = 5). *, *p* < 0.05.

To determine the in vivo effect of adjuvant CGN treatment after IR, we used a 4T1 xenograft animal model with the experimental schedule shown in Figure 6A. Tumor growth was significantly decreased in the group treated with CGN after IR compared to either CGN or IR treatment alone (Figure 6B). Similarly, the terminal tumor size at day 25 was smallest in the CGN and IR group (Figure 6C). Local invasion is the initial step in the spread of cancer cells from a local site to distant metastasis sites [30]. Therefore, we next determined the therapeutic effect of CGN in combination with IR on distant metastasis. Compared to the other groups, combined treatment with IR and CGN significantly inhibited lung metastasis (Figure 6D). We then confirmed RacGAP1 expression within tumors. Consistent with the in vitro data shown in Figure 5, RacGAP1 was suppressed in 4T1 tumors treated by either CGN alone or CGN in combination with IR (Figure 6E). Taken together, these results indicate that adjuvant CGN treatment effectively suppresses primary tumor growth and reduces metastatic potential, which may be the result of RacGAP1 suppression.

## 3. Discussion

In this study, we tested CGN as an adjuvant therapy to improve the effectiveness of radiotherapy. Administration of CGN to IR treatment increased cancer cell death. Furthermore, CGN treatment resulted in notable suppression of IR-induced cancer cell invasion. RacGAP1 signaling is a possible molecular mechanism of CGN effect after IR treatment on cancer cells. In the 4T1 xenograft model, combined treatment of IR and CGN significantly suppressed the tumor growth and lung metastasis.

Although local radiotherapy improves cancer treatment outcomes, recurrence or distant metastases following local treatment remain major therapeutic challenges. Local recurrences or distant metastases could be partially due to the enhancement of invasive properties in surviving cancer cells after IR [33]. We and others have shown that integrins are involved in the acquisition of cancer cell invasion after IR [34,35,40]. Several studies have reported that α5β1-integrin trafficking regulates RacGAP1 activation, which is essential to promote pseudopod extension and cancer cell invasion [19,38]. Here, we show that upregulation of RacGAP1 after IR is accompanied by increased invasion activity, and depletion of RacGAP1 significantly suppressed IR-induced cancer cell invasion (Figure 4). Moreover, CGN suppresses IR-induced invasiveness, which is partially restored by overexpression of RacGAP1 (Figure 5). These findings suggest that upregulation of RacGAP1 in cancer cells after IR treatment may be one of the pivotal mechanisms contributing to IR-induced invasiveness. The detailed regulatory mechanisms of RacGAP1 on IR-induced invasion should be investigated in future studies.

Previous studies suggest that RacGAP1 is a potential therapeutic target for the treatment of highly aggressive cancers. The clinical significance of RacGAP1 has been widely reported, and its expression in tumors is associated with more aggressive phenotypes in many cancers, including high-grade breast cancer, epithelial ovarian cancer, gastric cancer, colorectal cancer, and hepatocellular carcinoma in the transition from low- to high-invasive disease [15,20,39,41,42]. Besides, RacGAP1 is implicated in the resistance to doxorubicin treatment in squamous cell carcinoma [43]. To our knowledge, this is the first report to connect RacGAP1 to radiation resistance. In addition, we reveal a novel method for targeting RacGAP1 by administering CGN after radiation therapy, which may lead to future clinical application.

Besides RacGAP1, other genes were also found to be upregulated by IR and suppressed by the following CGN treatment (Figure S5). Within these genes, *AKAP9*, *CENPE*, *PRKCI*, *RDX*, *RECQL*, and *USO1* have also been reported to be associated with cancer progression [44,45,46,47,48,49,50,51,52,53,54,55]. *AKAP9* (encodes A-kinase anchor proteins-9) is involved in the development of metastasis of several cancers, including colorectal cancer [44], breast cancer [45], lung cancer [46], melanomas [47], thyroid carcinomas [48]. Inhibition of *CENPE*-encoded protein CENPE (Centromere-associated protein E), a kinetochore-associated mitotic kinesin, has been shown to induce cancer cell apoptosis and tumor regression [49]. *PRKCI* (encodes protein kinase C, iota) is overexpressed in ovarian cancer [50] and was suggested to promote immune suppression [51]. *RDX* (encodes radixin) is overexpressed in many tumor tissues and was suggested to enhance colon cancer cell invasion [52]. *RECQL*-encoded RecQ helicase-like protein is a DNA helicase which plays a vital role in the DNA damage response, and the mutation of *RECQL* has been suggested as a plausible candidate breast cancer susceptibility gene [53]. Knockdown of *USO1* (encodes general vesicular transport factor p115) was shown to inhibit cell proliferation and induce cell apoptosis in multiple myeloma cells [54] and colon cancer cells [55]. Although these molecules have various functions in cancer cells, they may be involved in radioresistance or radiation-induced invasion that can be targeted by CGN adjuvant treatment. Roles of these molecules and related mechanisms could be investigated in the future.

In our study, an increase in the proportion of polyploid cells was noted after the combined treatment with IR and CGN. Polyploid cells are considered the result of enhanced mitotic catastrophe [27,56]. Eriksson et al. revealed that IR treatment leads to a dose-dependent induction of morphological mitotic catastrophes and polyploid formation [28], which is accompanied by delayed DNA damage from IR [26]. The proportion of polyploid cells in HeLa Hep2 cells increases from 2.8 ± 1.3% to 17.6 ± 2.1% following treatment with 10 Gy [28]. Our data show that the proportion of polyploid cells in MDA-MB-231 cells was 5.6 ± 2.7% in the untreated group, 12.9 ± 1.7% in the CGN group, 17.6 ± 3.3% in the 4 Gy IR group, and 26.1 ± 1.9% in the IR and CGN group. Our results show that IR followed by CGN led to a higher proportion of polyploid cells, indicating that CGN could enhance the induction of cellular mitotic catastrophe following IR treatment. Aside from CGN, a variety of anticancer drugs are also known to induce mitotic catastrophe by influencing the stability of microtubule spindles or defective cell cycle checkpoints [57]. Mansilla et al. reported the result of mitotic catastrophe caused by the chemotherapeutic agent doxorubicin and the anthracycline antibiotic WP631 in MDA-MB-231 cells and MCF-7/VP cells [58]. In their study, treating cells with both agents resulted in increased polyploid formation, followed by increased cell death. The activation of the caspase-3-related apoptotic pathway was only observed in MDA-MB-231 cells treated with doxorubicin, indicating that caspase is not mandatory for cell death induced by mitotic catastrophe. In our study, cells cultured with CGN increased caspase-3 activity as well, suggesting that different patterns of cell death are also caused by CGN.

In Figure 6, single IR or CGN failed to control the tumor growth and metastasis in vivo. In this mouse model, we chose a low dose of IR treatment on a big tumor (about 500 mm^3^) to elucidate the adjuvant effect of CGN with IR. IR was treated from day 12 after injection of 4T1 cells. In fact, on day 12, the tumor size is already too big to be affected by 2 Gy × 4 dosages. In our preliminary experiments, when we treat the mice with higher doses (2 Gy × 5) on the smaller tumor (less than 100 mm^3^), we observed the effect by IR alone as compared with the untreated group. Several studies have reported the utilization of CGN in cancer immunotherapy. CGN promotes dendritic cell maturation through toll-like receptor 4 signaling [13]. CGN-treated dendritic cells significantly inhibited the tumor growth of murine lung tumor TC-1 compared with the control group [13]. In addition, a previous report showed that intratumoral injection of CGN decreases tumor growth in the B16-F10 or 4T1 tumor model in vivo by stimulating immune responses [11]. They treated with CGN every two days in the early stage of the subcutaneous 4T1 tumor growth, which decreased significantly 25 days after tumor inoculation in the CGN-treated group compared to the control group. In contrast, we showed that CGN treatment alone did not inhibit in vivo 4T1 tumor growth (Figure 6B), while it decreased cell viability in vitro (Figure 1A). We started CGN injection in the late stage of tumor growth and limited the treatment to three injections to better assess the adjuvant effect of CGN for radiation therapy. Therefore, the different in vivo results for CGN alone may be due to the differences in starting time and a total number of CGN injections. It has also been reported that radiation affects immune cells surrounding the irradiated tumors [59], but its role in either stimulating or dampening anti-tumor responses is not fully understood. To effectively induce the immune response by CGN combined with IR, the treatment condition should be optimized. Further investigation into the alteration of infiltrating immune cell subsets following treatment with IR and CGN may lead to a better understanding of their combined cytotoxic effects.

Carrageenan has been considered to cause inflammatory events of the gastrointestinal tract, which may limit the utilization to use in clinical treatment. Early studies reported that CGN administered in drinking water or diet could cause intestinal inflammation and ulcers in animals [60,61]. This phenomenon has been exacerbated in animal studies where CGN injected into confined spaces in an animal’s body, such as the hind paw, pleural space, or peritoneal cavity [62,63]. However, safety studies conducted over the last 15–20 years in which CGN was administered to test animals through the diet have not shown any adverse effects [64]. These conflicting results are thought to be due to the difference in the purity of “carrageenan” [65]. McKim et al. found that commercial CGN can be diluted with sugars (dextrose or sucrose) and, thus, the potential to inadvertently add contaminants, such as bacteria, is high. In our study, the purity of CGN we used is 88.72 ± 2.21%, which is higher than the previous study used [65]. On the other hands, the amount of glucose was 4.23 ± 0.62% of the initial weight. These results suggest that high purity CGN which we used would be difficult to cause a pro-inflammatory effect.

In our study, the expression of RacGAP1 was found to be significantly suppressed by CGN treatment. Although the detailed mechanism is still unclear, other studies suggest possible pathways. For instance, Signal transducer and activator of transcription 3 (STAT3) is a transcriptional factor which has been reported to activate the transcription of RacGAP1 in hepatocellular carcinoma cells [42]. Several studies reported that some polysaccharides from plants could induce biological effects in cells via suppression of STAT3-related pathways [66,67]. Therefore, it is reasonable to the hypothesis that CGN, as a natural polysaccharide, regulates the expression of RacGAP1 via STAT3-related pathway. These studies may provide us the clues about the mechanism of how CGN regulates RacGAP1, which are worth further investigation.

Taken together, our results suggest a possible therapeutic strategy involving CGN treatment as an adjuvant to radiotherapy for the suppression of tumor growth and the reduction of distant metastasis.

## 4. Materials and Methods

### 4.1. Cell Culture

MDA-MD-231 human breast cancer cell line (ATCC^®^ HTB-26™), PANC-1 human pancreatic cancer cell line (ATCC^®^ CRL-1469™), FaDu human head and neck cancer cell line (ATCC^®^ HTB-43™) and 4T1 mouse mammary carcinoma cell line (ATCC^®^ CRL-2539™) were purchased from American Type Culture Collection (ATCC; Manassas, VA, USA). MDA-MB-231 and PANC-1 cells were cultured in Dulbecco’s modified Eagle’s medium (DMEM; Nacalai Tesque, Kyoto, Japan) containing 10% fetal bovine serum (FBS; HyClone, GE Healthcare Life Sciences, Logan, UT, USA). FaDu cells were cultured in minimum essential medium eagle (Sigma-Aldrich, St Louis, MO, USA) containing 10% FBS. 4T1 cells were cultured in RPMI-1640 (Sigma-Aldrich) containing 10% FBS. 

### 4.2. Carrageenan

Lambda-carrageenan plant mucopolysaccharide (Sigma-Aldrich, Lot number BCBP8978V) was dissolved in Milli-Q water at a concentration of 10 mg/mL. The typical molecular weight of λ-carrageenan was reported as 1054 kDa [68]. The purity of carrageenan used in this study was 88.72 ± 2.21%, which was determined by EDTA/2-propanol recovery method [65]. The amount of glucose/dextrose dissolved in the wash solution was also determined by Picoprobe Glucose Assay Kit (Abcam, Cambridge, UK), and the results suggest that 4.23 ± 0.62% of the initial weight is accounted by glucose. To dissolve CGN in water, the solution of CGN was gently shaking for 24 h in 37 °C. And then, CGN was filtered through 0.45 µm filters (Advantec, Tokyo, Japan). Cells were treated with 2.5 mg/mL CGN or Milli-Q water for 24 h after 4 Gy IR treatment.

### 4.3. Irradiations

Cells were irradiated with 4 Gy 130 kV X-rays using a CellRad X-ray generator (CellRad; Faxitron, Tucson, AZ, USA). Mice were irradiated with a daily fraction of 2 Gy 125 kV X-rays for four days (HITACHI).

### 4.4. Cell Viability Assay

Cells were seeded and treated with 4 Gy IR at 50–60% confluency. Twenty-four hours after IR, cells were treated with 2.5 mg/mL CGN (approximately 2.2 mg/mL CGN is contained considering purity mentioned above) or Milli-Q water for 48 h and then subjected to each experiment. Cell viability and cytotoxicity were examined by cell counting using the trypan blue exclusion method and flow cytometry after PI staining. The proportion of PI-positive cells was quantified by placing polygon gate.

### 4.5. Apoptosis Analysis

Annexin V staining was performed using an Annexin V-FITC Apoptosis Detection Kit (Abcam). Cells more than 1 × 10^5^ were harvested, resuspended in 500 µL binding buffer, and incubated with Annexin V-FITC and PI for 5 min at room temperature in the dark. Fluorescence was analyzed using a FACSAria III flow cytometer (BD Biosciences, Franklin Lakes, NJ, USA). Mean fluorescence intensity of FITC was calculated and normalized to the untreated group.

To determine caspase activity, caspase-3, caspase-8, and caspase-9 multiplex activity assay kit (Abcam) was used. Briefly, cells were treated and seeded in 96-well plates at 2 × 10^4^ cells/100 µL FACS buffer (2% FBS in PBS). After incubation at 37 °C, 5% CO_2_ for 1 h, fluorescence was monitored using a microplate reader (CLARIOstar; BMG LABTECH, Ortenberg, Germany) with the following wavelengths: caspase-3 excitation (Ex)/emission (Em) = 535/620 nm; caspase-8 Ex/Em = 490/525 nm; caspase-9 Ex/Em = 370/450 nm. 

### 4.6. ROS Detection Assay

To measure ROS levels in cells, treated cells were stained with 20 µM dichlorofluorescein diacetate (DCFDA) for 30 min at 37 °C using a Cellular ROS detection assay kit (Abcam). Cells were then analyzed using a FACSAria III flow cytometer.

### 4.7. DNA Content and Polyploidy Analysis

For polyploidy analysis, 70% of ethanol was added slowly to cell pellets. Cells were stored at -80 °C overnight, and then cells were centrifuged and washed with cold PBS two times. Cells were then resuspended in 300 µL staining solution (0.1% (v/v) Triton X-100, 2 mg RNase A (NIPPON GENE, Tokyo, Japan) and 400 µL of 500 µg/mL PI (Setareh Biotech, Eugene, OR, USA) in 10 mL PBS. After incubation at 37 °C for 15 min, samples were analyzed by a FACSAria III flow cytometer.

### 4.8. Immunofluorescence

Cells were fixed in 4% paraformaldehyde (PFA), permeabilized with 0.2% Triton X-100/PBS, and then washed with PBS. For examination of polyploidy, cells were incubated with an α-tubulin (Cell Signaling Technology, Danvers, MA, USA) antibody after blocking, and then washed with PBS, followed by incubation with an Alexa Fluor secondary antibody. Cell nuclei were counterstained with PI. For images of RacGAP1 localization, cells were stained with an anti-RacGAP1 antibody (Abcam), an α-tubulin antibody and DAPI. Images were acquired by Leica True Confocal Scanning (TCS) SP8 microscope system (Leica Microsystems, Wetzlar, Germany).

### 4.9. Matrigel Invasion Assay

The Matrigel chemoinvasion assay was performed using Biocoat Matrigel invasion chambers (Corning Inc., Corning, NY, USA) or 24-well hanging inserts 8.0 μm PET Millicell cell culture inserts (Merck Millipore, Darmstadt, Germany) coated with Matrigel growth factor reduced (GFR) basement membrane matrix (Corning Inc.). For coating Millicell inserts, 100 μL serum-free medium containing 400 μg/mL Matrigel was evenly distributed on the membrane of the Millicell insert chamber, followed by incubation at 37 °C for at least 2 h. During the invasion assay, cells suspended in the DMEM with 0.1% BSA were seeded on the upper chambers, and the lower wells were filled with DMEM with 10% FBS. After incubation for 8 h, cells that migrated out onto the lower surface of the membranes were fixed in 4% PFA and stained with 1% crystal violet. Data were collected from four independent experiments and normalized to the results of the untreated group.

### 4.10. Western Blotting

Western blotting was performed as described previously [35]. Briefly, cell lysates were separated by SDS-PAGE or Nu-PAGE Bis-Tris protein gels (Thermo Fisher Scientific, Waltham, MA, USA), and then transferred onto a polyvinylidene fluoride (PVDF) membrane (Merck Millipore), and then blocked with Odyssey^®^ blocking buffer (LI-COR Biosciences, Lincoln, NE, USA). Membranes were probed with primary antibodies, anti-RacGAP1 (Proteintech, Rosemont, IL, USA or Abcam) or anti-β-actin (Sigma-Aldrich, St Louis, MO, USA), and then washed with Tris-buffered saline Tween-20 (TBST). The membranes were incubated with secondary antibodies and then washed with TBST. The signals were detected with an Odyssey CLx Imager (LI-COR Biosciences). 

### 4.11. Microarray Analysis

After treatment with CGN and IR, total RNA of MDA-MB-231 cells was isolated using a NucleoSpin^®^ RNA kit (MACHEREY-NAGEL, Düren, Germany). For the microarray analysis, the high sensitivity 3D-Gene^®^ Human oligo chip 25k version 2.10 (Toray Industries, Tokyo, Japan) was used. The data were normalized and analyzed by Toray Industries. Gene expression values lower than 100 after global normalization were excluded. To focus on the effects of CGN, genes were selected by the following order (Figure S1). First, genes increased 1.25-fold or higher in the IR group compared to the untreated group were selected. Second, genes reduced to one-eighth or less in IR and CGN treatment groups, compared to untreated, were selected. The genes that meet both of these criteria were selected as candidates for further analyses.

### 4.12. siRNA and Transfection

To knockdown RacGAP1, siRNAs with the following sequences were used: negative control: 5′-GUUUAUUGACAAGUUAAGAdTdT-3′ (sense), 5′-UCUUAACUUGUCAAUAAACdTdT-3′ (antisense); siRacGAP1 # 1: 5′-CAGGUGGAUGUAGAGAUCAAAdTdT-3′ (sense), 5′-UUUGAUCUCUACAUCCACCUGdTdT-3′ (antisense); siRacGAP1 # 2: 5′-CUAGGACGACAAGGCAACUUUdTdT-3′ (sense), 5′-AAAGUUGCCUUGUCGUCCUAGdTdT-3′ (antisense). The siRNA duplexes were synthesized by Hokkaido System Science. Cells were transfected with siRNA duplexes using Lipofectamine RNAiMAX (Thermo Fisher Scientific).

### 4.13. Overexpression

For RacGAP1 overexpression, complementary DNA (cDNA) of RacGAP1 was obtained by PCR from the first-strand cDNA of MDA-MB-231 cells. The RacGAP1 cDNA was subcloned into the mVenus N1 vector and followed by subcloning into a PiggyBac transposon-based doxycycline-inducible vector, pPB-TRE3G-MCS-CEH-rtTA3-IP [69]. Transfection of the resulting RacGAP1-mVenus plasmid, together with a hyperactive PiggyBac transposase vector [69] to MDA-MB-231 cells was performed by ViaFect transfection reagent (Promega, Madison, WI, USA), and cells were selected by puromycin. The expression of RacGAP1-mVenus was induced by the addition of 200 ng/mL doxycycline.

### 4.14. In Vivo Study 

One million 4T1 cells in 100 μL PBS were injected into the left thighs of six-week-old Balb/c mice subcutaneously. Eleven days after inoculation, the mouse tumors were treated with a daily fraction of 2 Gy of X-rays for four days under anesthesia. Tumors were treated with 50 mg/kg CGN or PBS 3 times by intratumoral injection one week after the first IR fraction. Tumor size was measured after radiation and normalized to the tumor size measured on the first day after radiation. All animal procedures were approved by the Institutional Animal Care and Use Committee of Hokkaido University (# 16-0137).

### 4.15. Immunohistochemistry

The tumor tissues were collected and placed in 4% PFA solution, fixed for 24 h, dehydrated through a gradient of ethanol, and embedded into paraffin blocks for immunohistochemistry. The paraffin blocks were cut to 4 µm sections and mounted onto microscope slides for analysis. For antigen retrieval, the slides of tumor sections were incubated with antigen unmasking solution (Vector Laboratories, Burlingame, CA, USA) at 80 °C for 1 h. Endogenous peroxidase activity was quenched using 3% H_2_O_2_ in 10% methanol. Each slide was incubated in 2% blocking buffer (Roche, Basel, Switzerland) for 1 h, and then incubated with a RacGAP1 primary antibody (Proteintech) overnight. Super Sensitive IHC Detection Systems (BioGenex, Fremont, CA, USA) were used to amplify the signal. Sections were stained with horseradish peroxidase (HRP) secondary antibodies. After two washes, the slides were counterstained with hematoxylin (Muto Pure Chemicals, Tokyo, Japan). Positive staining was scored using the following formula: (r3/t) × 3 + (r2/t) × 2 + (r1/t) × 1, where t is the total area of tumor tissue for a whole tumor section, r3 is the total area of high-intensity staining (intensity 3), r2 is the total area of medium intensity staining (intensity 2), and r1 is the total area of weak intensity staining (intensity 1).

### 4.16. Statistical Analysis

All in vitro results were confirmed by at least three independent experiments. Data were analyzed by two-tailed Student’s *t*-tests. Graphs are presented in columns as mean values ± standard error of the mean (SEM). For in vivo experiments (*n* = 6–7 in each group), normality of the data sets was examined by the Kolmogorov-Smirnov test, where *p* > 0.05 state in a normal distribution. After the judgment of the equality of variance by the F-test, statistical significance was examined by the two-tailed *t*-test for equal variance or *t*-test with Welch correction for unequal variances. For data with non-normal distribution, statistical significance was examined by the Mann-Whitney test after confirming equal variance by the F-test. Graphs are presented in scatter plots. Significant differences are indicated by * *p* < 0.05, ** *p* < 0.01, *** *p* < 0.001 and n.s. for not significant.

## 5. Conclusions

In this study, we found that RacGAP1 expression is increased after irradiation and associated with cancer cell invasion. We also found that CGN treatment following radiotherapy effectively suppressed the expression of RacGAP1 in in vitro cell culture and in vivo mouse tumor model. Based on these data, we conclude that CGN enhances the effect of radiotherapy by suppressing cancer cell survival and invasiveness through the RacGAP1 pathway. We propose the novel application of CGN as an adjuvant for radiotherapy in clinical use.

## Supplementary Materials

The following are available online at https://www.mdpi.com/2072-6694/11/8/1192/s1, Figure S1: Analysis of Annexin V/PI double staining. Figure S2: The effect of CGN and/or IR in normal cell line, MCF10A. Figure S3: Caspase-3 activity measured by western blot. Figure S4: Cell viability during the Matrigel chemoinvasion assay. Figure S5: Gene expression profiles after IR and CGN treatments. Figure S6: Immunofluorescence staining of siRNA-mediated knockdown of RacGAP1 in MDA-MB-231cells. Figure S7: Uncropped scans of western blots.
