# Supplementary material for: Lambda-Carrageenan Enhances the Effects of Radiation Therapy in Cancer Treatment by Suppressing Cancer Cell Invasion and Metastasis through Racgap1 Inhibition
Source: Cancers (Basel). 2019 Aug 16;11(8):1192. doi: 10.3390/cancers11081192 (PMC6721563; doi:10.3390/cancers11081192)
Supplement: Supplementary file 1 [file cancers-11-01192-s001.pdf]

Supplementary Materials

Lambda-carrageenan enhances the effects of radiation therapy in cancer treatment by suppressing cancer cell invasion and metastasis through RacGAP1 inhibition

Ping-Hsiu Wu, Yasuhito Onodera, Frances C. Recuenco, Amato J. Giaccia, Quynh-Thu Le, Shinichi Shimizu, Hiroki Shirato and Jin-Min Nam*

1. Supplementary Materials and Methods

1.1. Cell Culture

MCF10A human epithelial cell line (ATCC ® CRL-10317™) were purchased from American Type Culture Collection (ATCC; Manassas, VA, USA). MCF10A cells were cultured in DMEM/F12 (Thermo Fisher Scientific, Waltham, MA, USA) supplemented with 5% horse serum (Thermo Fisher Scientific); 20 ng/ml epidermal growth factor (EGF; Roche, Basel, Switzerland); 10 μg/ml insulin (Sigma-Aldrich, St Louis, MO, USA); 0.5 μg/ml hydrocortisone (Sigma-Aldrich); 100 ng/ml cholera toxin (Sigma-Aldrich) and Penicillin-Streptomycin (Thermo Fisher Scientific).

1.2. Cell Viability Assay during Invasion Assay

Cell viability during invasion assay was determined using the Cell Counting Kit-8 (CCK-8; Dojindo). The same number of cells in each group during invasion assay was seeded into a 96-well plate. When invasion assay was finished, CCK-8 solution was added to cells in 96-well plates for 1 h at 37 °C. To measure cell viability, absorbance at 450 nm was determined using a Multiskan™ GO Microplate Spectrophotometer (Thermo Fisher Scientific).

1.3. Western blotting

To measure active caspase-3, western blot was performed using anti-cleaved caspase-3 antibody (Abcam) and anti-β-actin (Sigma-Aldrich).

2. Supplementary Figures

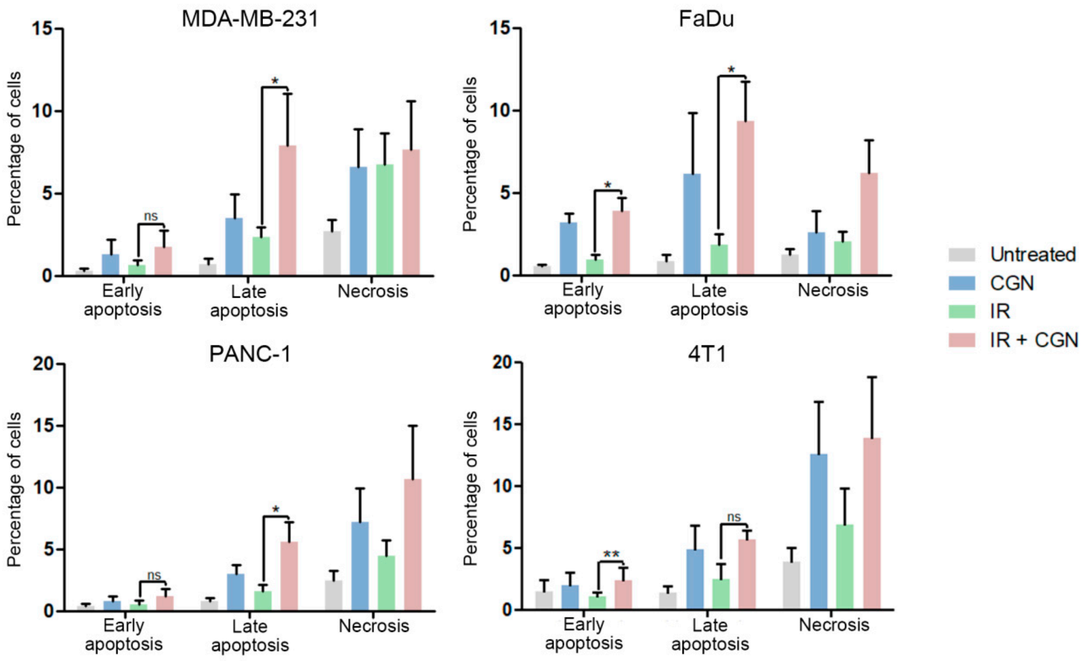

Figure S1. Analysis of Annexin V/PI double staining. The percentage of early apoptotic cells (Annexin V-positive, PI-negative), late apoptotic cells (Annexin V-positive, PI-positive) and necrotic cells (Annexin-V negative, PI-positive) followed by flow cytometry in MDA-MB-231, FaDu, PANC-1, and 4T1 cells. Columns, mean ($n \geq 3$); bars, SE. *, $p < 0.05$, **, $p < 0.01$; ns, not significant.

.

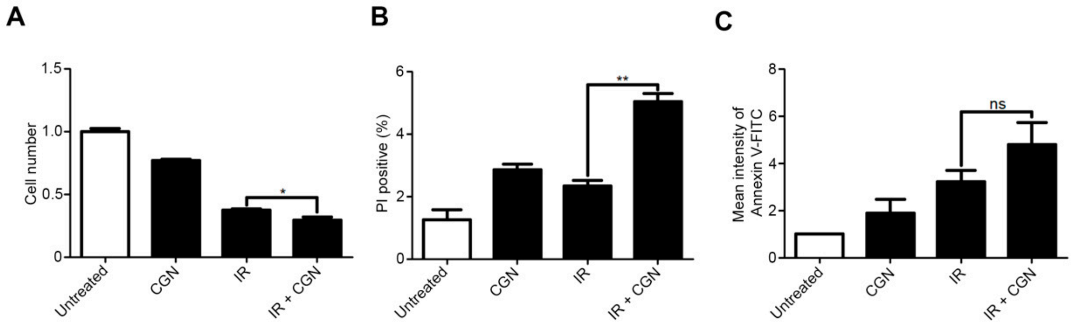

.**Figure S2.** The effect of CGN and/or IR in normal cell line, MCF10A. Cells were treated with 4 Gy IR, followed by CGN on the next day, and then analyzed 72 h after IR. (**A**) Cell viability was quantified by cell counting in MCF10A cell lines. (**B**) The percentage of dead cells was measured by PI staining, followed by flow cytometry. (**C**) Apoptotic cells were measured Annexin V-FITC staining, followed by flow cytometry. Columns, mean (n = 3); bars, SE. *, $p < 0.05$; **, $p < 0.01$; ns, not significant.

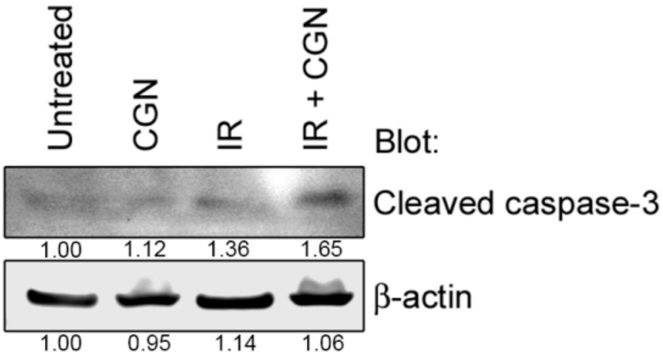

Figure S3. Caspase-3 activity measured by western blot. Lysates of MDA-MB-231 cells were blotted with anti cleaved caspase-3 antibody.

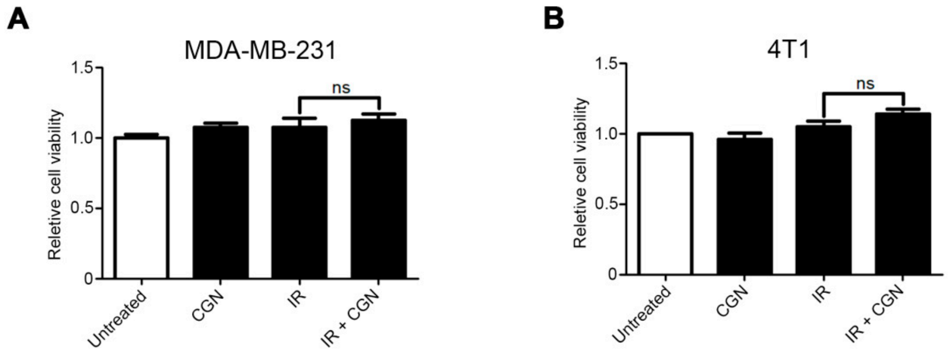

Figure S4. Cell viability during the Matrigel chemoinvasion assay. (**A, B**) Viability of MDA-MB-231 (**A**) and 4T1 cells (**B**) was measured by Cell Counting Kit-8 (CCK-8) after 0 Gy or 4 Gy IR treatments with or without CGN. Data were collected from three independent experiments with duplicate, and normalized to the untreated group. Columns, means (*n* = 3); bars, SE. ns, not significant.

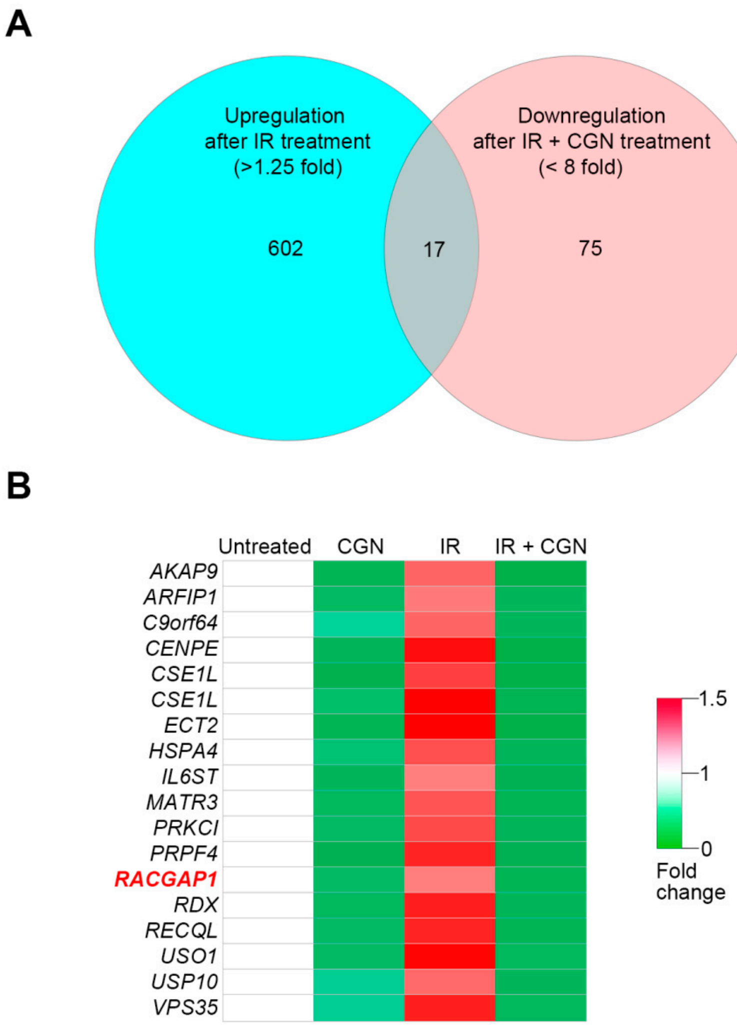

Figure S5. Gene expression profiles after IR and CGN treatments. (**A**) Venn diagram shows the number of genes upregulated by IR treatment and downregulated by IR and CGN treatments compared to untreated. Of the 24,462 probes on the cDNA microarray, 619 genes were upregulated (> 1.25 fold) after IR treatment, and 92 genes were downregulated (< 8 fold) after IR and CGN treatments compared to untreated. The 17 genes included in the overlapping intersect were selected. (**B**) List and heat map of the 17 selected genes that are upregulated by IR (red) and downregulated by IR and CGN treatments (green) compared to untreated. The values in each group were normalized to the untreated group.

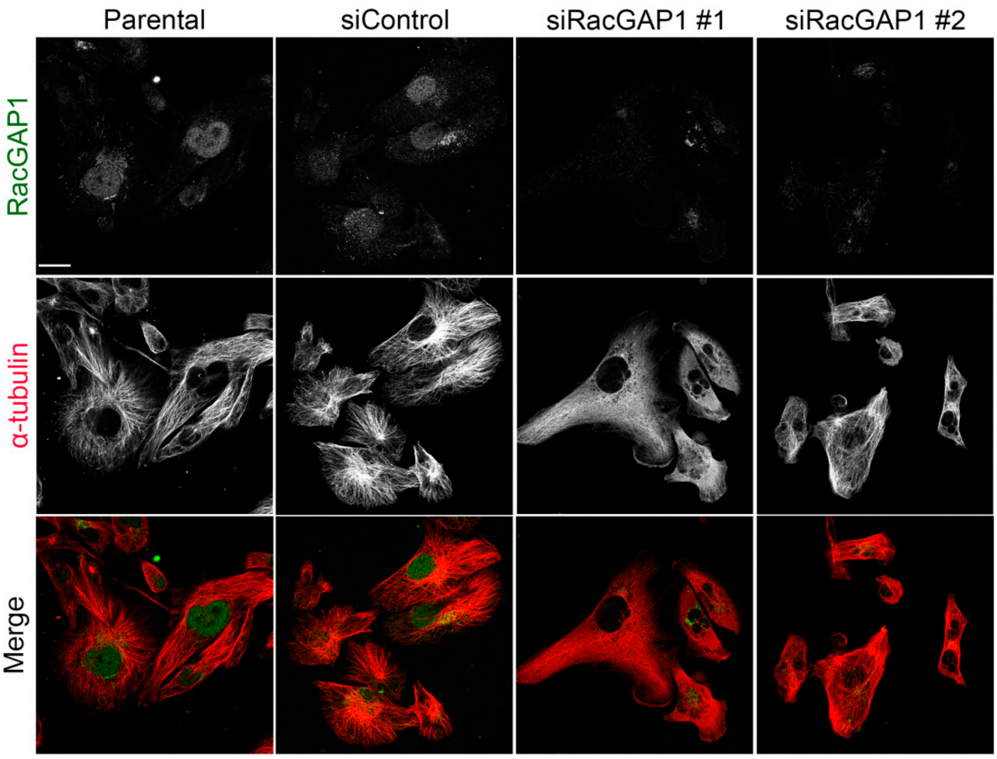

Figure S6. Immunofluorescence staining of siRNA-mediated knockdown of RacGAP1 in MDA-MB-231cells. RacGAP1 (green) and α-tubulin (red). Bar, 25 μm.

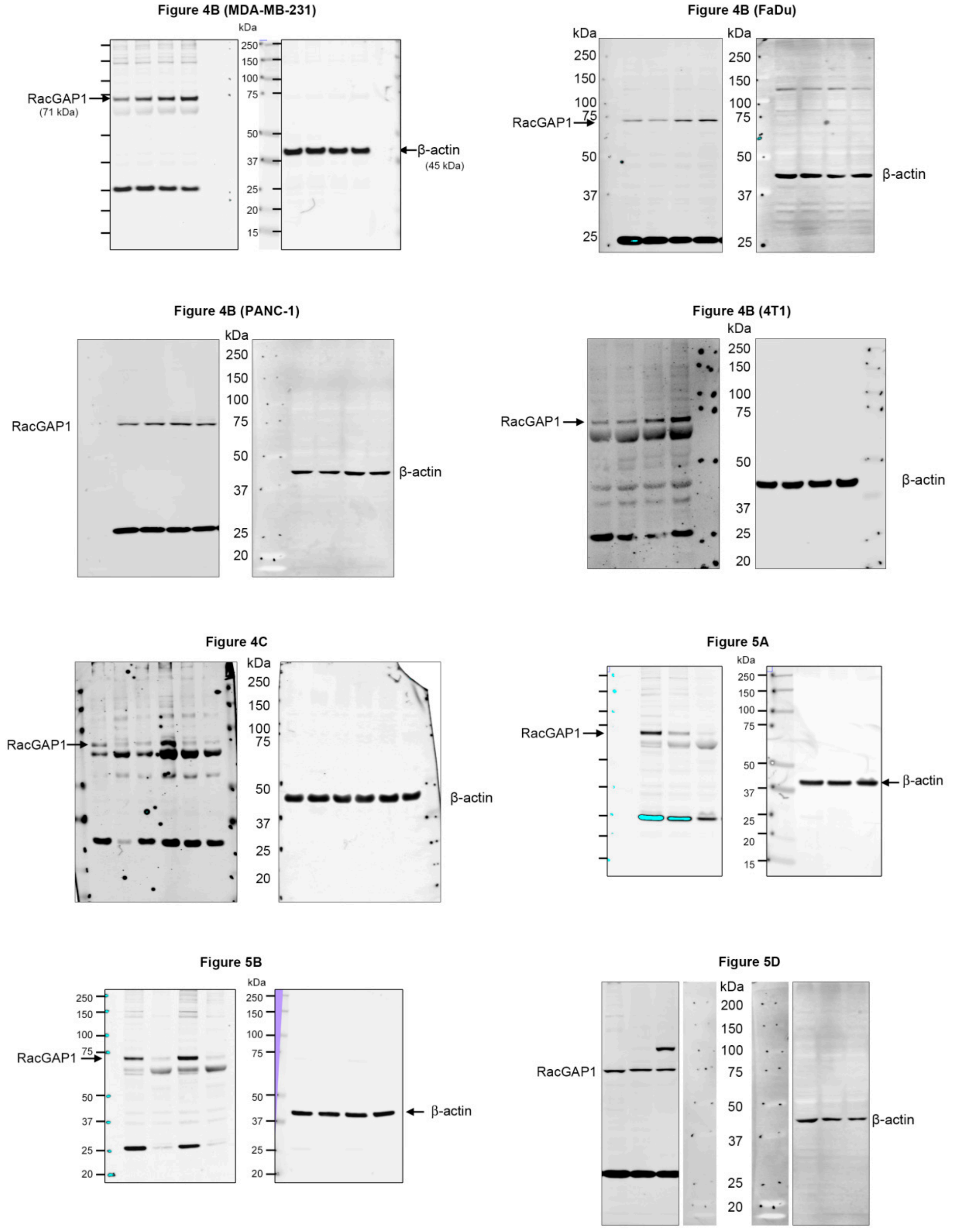

Figure S7. Uncropped scans of western blots.
